# Rapid assessment of COVID-19 suspected cases: A community based approach for developing countries like Pakistan

**DOI:** 10.7189/jogh.10.010353

**Published:** 2020-06

**Authors:** Naeem Shahzad, Irfan Abid, Wajahat Javed Mirza, Muhammad Mazhar Iqbal

**Affiliations:** 1 National University of Sciences and Technology, Risalpur Campus, Pakistan; 2 Rawalpindi Institute of Cardiology, Rawalpindi, Pakistan

The Coronavirus pandemic started in late December 2019, when an unexplained case of mass pneumonia occurred in Wuhan, China raising concern of the responsible health department of the City. The Chinese government notified the WHO of the epidemic situation in the first week of January 2020 and subsequently, the causative agent was identified as a new coronavirus (2019-nCoV), followed by genetic sequence analysis and the development of a detection method [1]. In the second week of January, the new coronavirus pneumonia was included in the management of Class B infectious diseases by the National Health and Health Commission China on the approval of the State Council. City went under lockdown and the Chinese government made the highest-level commitment to mobilize every effort to stop the epidemic. Understanding the epidemiological characteristics of Corona Virus (2019-nCoV) transmission is critical to developing and implementing effective control strategies. This virus spread rapidly affecting 31 provinces (autonomous regions / municipalities) from 30 days after the first reported case, the epidemic reached its first peak on January 24-26, 2020, and an unusually high incidence of single-day event on February 12, 2020, and then gradually decreased till China was able to contain this virus to its track [2].

COVID-19 has become one of the largest spreading diseases on the global front in the recent times after Spanish flu in early 20^th^ century. Current globalized environment and rapid modes of transportation have helped spreading this virus at an enormous rate, which is unprecedented. The non-availability of cure for the disease and rapid transfer rate has brought the world to a halt. There is also a deficiency of testing kits on the global front coupled with the capacity of available health facilities to test and treat the patients, especially in the under developed world. This required a scientific approach to shortlist the case requiring medical attention for testing and treatment. Another important factor raising red flag for the spread is a period of 14 days inactivity of the virus, which may lead to a wrong diagnostic, resulting into spread of the disease.

Current global outbreak of Novel Corona Virus has highlighted the inherent gaps in preparedness level and management of the biological disasters including the most developed nations of the world. Despite inclusion of epidemics and pandemics amongst biological hazards by the Sendai Framework for Disaster Risk Reduction (UNISDR, 2015) and other related frameworks and conventions, the Corona Virus outbreak has exposed the loop holes which many countries are struggling to respond and manage, as their health care systems have been overwhelmed by exponential increase in cases with every passing day. It is worth mentioning that notwithstanding all odds, countries like China, South Korea and Singapore have effectively controlled the spread of this pandemic while US is managing huge number of patients with considerable fatalities.

**Figure:**
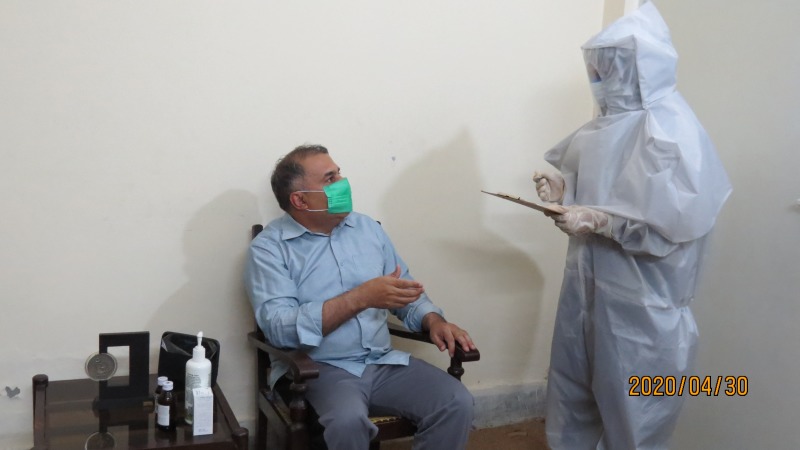
Photo: Nurse wearing locally produced low cost COVID-19 kit and filling the score card from a suspected patient for initial screening and triage at the emergency control room (from the collection of Dr Naeem Shahzad, used with permission).

By observing the response of the US and most European countries to COVID-19, it is apparent that the developing countries and countries with poor economic infrastructure will not be able to cope up with this health emergency. It is contingent that countries with low GDPs are already over burdened due to number of factors including overwhelmed health care systems and different underlying co-morbidities. Therefore, it is imperative to infer that risks posed by COVID-19 in countries with poor economies will be entirely different as compared to US, China and Europe. The average household in low-income countries is on higher side as compared to middle and high-income countries thereby highlighting the vulnerabilities of these countries to rapid spread of COVID-19[3]. For this reason depending on the existing health care systems in countries with low GDPs will be unrealistic to contain and stop the COVID-19 transmission.

Different strategies for suppression of the COVID-19 are being practiced globally ranging from lockdown of cities to isolation of cases, in order to prevent the number of cases having severe illness requiring ICU [4]. Different countries are resorting to massive lock down and social distancing to manage and contain the COVID-19. This is more critical in the case of developing countries like Pakistan to avoid stretching of its already weak health care facilities by resorting to excessive testing, contact tracing, isolation and quarantine of the suspected cases to circumvent health care system overload [5]. Aggressive testing of the individuals becomes tiring and cumbersome in the absence of COVID-19 testing kits. Therefore, this study has made an effort to design a rapid assessment score card using bottom up approach starting from community level which will help the low-income and developing countries to ascertain the suspected COVID-19 cases at community level. This will be quite beneficial for rapid triage especially for the countries like Pakistan where the suspected cases of COVID-19 are likely to be under-detected due to low testing capabilities [6].

Based on different research findings [7-12], a score card has been developed encompassing maximum possible/ probable causes of COVID-19 suspicion among the community. To develop an effective tool for patient screening without overburdening the health care infrastructure while still not compromising the control over the spread of the disease and preventive measures, an effective score card has been developed covering numerous effective parameters for rapid assessment of the probable COIVD-19 cases, as shown in **Figure 1**. For this purpose the trends and effects of spread rate, age factor, previous health history, travel history, isolation period, appearance of the symptoms and contact with the suspected or confirmed patients has been taken into consideration. This score card has been designed for low income countries with no or limited testing capabilities of COVID-19 tests. It is perceived that countries like Pakistan, could resort to this score card at community level to triage the suspected patients and may adopt testing of only those patients who have higher scores (ie, ˃100). This will help lessen the burden of already limited laboratory testing facilities available in the country. The method will also be helpful for low-income countries like Afghanistan, Bangladesh, Chad etc., which are vulnerable to exponential outbreak of this virus due to their in-capacity to detect COVID-19 patients and availability of the testing kits is badly hampering and overburdening the WHO efforts to fight this pandemic.

**Figure 1:**
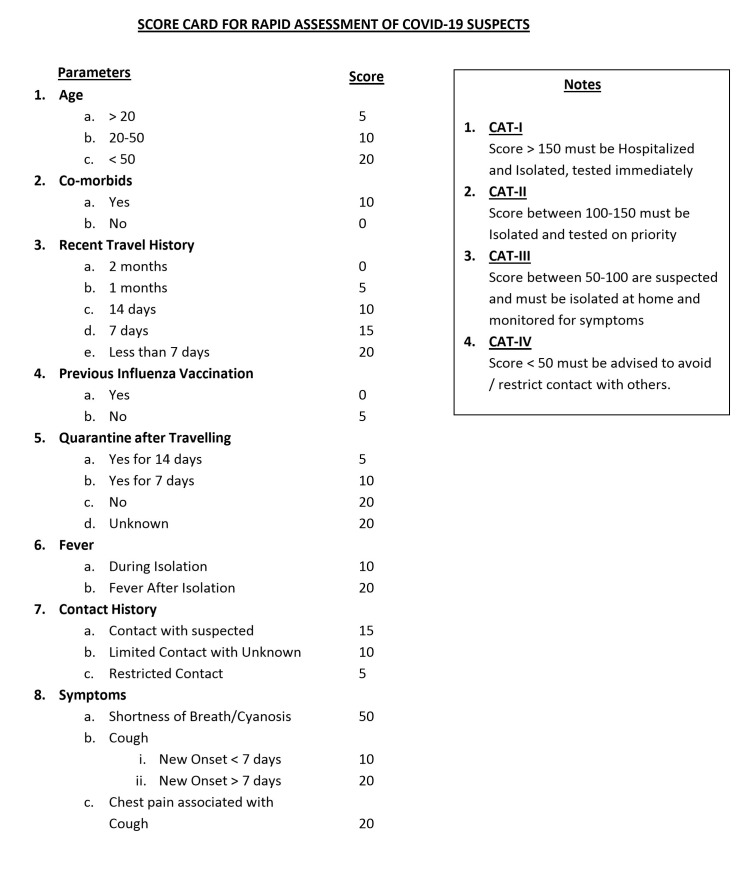
The proposed score card for rapid COVID-19 diagnosis.

The score card has been tested on a small community of Risalpur Cantonment (34.06048 latitude, 71.99276 longitude) which is an enclosed community consisting of a population of around 30 000. The demographic distribution of this community cannot be shared due to security issues as it is housed in a Cantonment. This score card was tested for its effectiveness after the area was under complete lock down since March 23, 2020 and limited entrance and exit was allowed to this community. An emergency control room has been developed in the community to deal with the COVID-19 situation. This room is manned 24/7 and any person entering Risalpur Cantonment was sent here in order to screen the individual for COVID-19 suspicion. Besides, any individual complaining or expecting probable symptoms of COVID-19 was first screened at this centre and was then further referred to the Hospital if required. Until April 3, 2020, 1166 persons were screened at the control room.

These results show that 21 persons were suspected and their scores suggested that they were required to be tested on priority, while 14 others were immediately hospitalized. Since the burden of patients was not much at the health facility available in the area, which is Class ‘C’ hospital, so tests of all the suspected patients ie, 35 were sent to Islamabad and Peshawar and all tested negative. However, these patients were isolated and quarantined for 14 days. It can be easily inferred that instead of testing all the 1166 individuals who reported to the control room for suspicion of COVID-19, only 35 persons required COVID-19 tests which accounts for 3% only. Low-income countries with limited health facilities may resort to this technique to reduce the burden on their testing facilities. It is pertinent to mention that this is not an ideal way of tracing the COVID-19 suspected patients but can be an alternative to No Testing at all due to minimal or low testing capacities.

Besides findings highlighted above, following deductions have been made for the observed statistics.

1. For a country where the disease has been imported after February 20, 2020, the rate of spread has been lower while still being on the exponential scale, as the preventive model of isolation and lockdown of the society was already inferred basing on the initial devastation of the disease.
2. Poor health facilities result in better immunity of the people to fight against the illness but the same also results in a less average age due to a fatigued life, there by having significantly less people with age over 70 years in the under developed world.
3. Previous health history is associated to age, health infrastructure, trends in the society, and to some extent geographical location. This also contributes significantly to the potential proneness for disease adaptability and seriousness.
4. Nevertheless, travel history is also a significant parameter. Although most of the countries have started to exercise the travel ban internationally, but the ghost phenomenon of the disease for a period of 14 days and local mobility within the country or affected region can still affect the patient count dramatically (both for confirmed and non-confirmed cases).
5. An already exiting mechanism evolved over the experience of last few weeks to deal with the spread, prevention, and cure of this disease has shown the efficacy of the isolation period and appearance of the symptoms of the disease. This is helpful in eliminating the disease and its spread in a locality or a region.

## CONCLUSIONS

The two successful strategies to contain and control Corona Virus have been adopted by China and Korea [13,14]. For countries like Pakistan, Korea’s strategy to Trace, Test and Treat is difficult to adopt due to limited testing capabilities. Similarly, owing to the poor economic conditions of the country, complete lock down will lead to economic crisis especially for small businesses and daily wagers. In addition to enhancing the country’s testing capabilities by importing test kits, it is imperative to adopt suggested score card based assessment, to reduce the load on the testing centers all across the country. This will be very beneficial in small villages and towns to adopt a bottom up approach and ease the already over stretched testing facilities for management, treatment and care of the suspected COVID-19 patients. Basing on the above mentioned factors, the over burdening of the health system can be controlled with a systematic filtering of the suspect cases, where the parameters discussed have been given due weightage to assess the total score of the suspect to filter him/her for a probable test case or otherwise.
